# Multi-Omics Analysis Reveals Novel Subtypes and Driver Genes in Glioblastoma

**DOI:** 10.3389/fgene.2020.565341

**Published:** 2020-11-26

**Authors:** Yang Yuan, Pan Qi, Wang Xiang, Liu Yanhui, Li Yu, Mao Qing

**Affiliations:** ^1^Department of Neurosurgery, West China Hospital, Sichuan University, Chengdu, China; ^2^Department of Dermatology, Chongqing Traditional Chinese Medicine Hospital, Chongqing, China; ^3^Department of Anesthesia, West China Hospital, Sichuan University, Chengdu, China

**Keywords:** multi-omics analysis, copy number variation, DNA methylation, mRNA expression, glioblastoma

## Abstract

Glioblastoma is the most lethal malignant primary brain tumor; nevertheless, there remains a lack of accurate prognostic markers and drug targets. In this study, we analyzed 117 primary glioblastoma patients’ data that contained SNP, DNA copy, DNA methylation, mRNA expression, and clinical information. After the quality of control examination, we conducted the single nucleotide polymorphism (SNP) analysis, copy number variation (CNV) analysis, and infiltrated immune cells estimate. And moreover, by using the cluster of cluster analysis (CoCA) methods, we finally divided these GBM patients into two novel subtypes, HX-1 (Cluster 1) and HX-2 (Cluster 2), which could be co-characterized by 3 methylation variable positions [cg16957313(DUSP1), cg17783509(PHOX2B), cg23432345(HOXA7)] and 15 (PCDH1, CYP27B1, LPIN3, GPR32, BCL6, OR4Q3, MAGI3, SKIV2L, PCSK5, AKAP12, UBE3B, MAP4, TP53BP1, F5, RHOBTB1) gene mutations pattern. Compared to HX-1 subtype, the HX-2 subtype was identified with higher gene co-occurring events, tumor mutation burden (TBM), and poor median overall survival [231.5 days (HX-2) vs. 445 days (HX-1), *P*-value = 0.00053]. We believe that HX-1 and HX-2 subtypes may make sense as the potential prognostic biomarkers for patients with glioblastoma.

## Introduction

Gliomas are most common malignant brain tumors which derive from neuroepithelial cells (Rivera et al., 2008). Most patients underwent tumor resection surgery with standard follow-up chemotherapy/radiotherapy, and based on molecular neuropathology diagnosis, they may survive from months to decades (median survival from 1 year to 15 years) (Marton et al., 2019). High-grade gliomas’ recurrence was due to their invasive nature. Recent studies on molecular pathology of glioma has outlined some valuable prognosis biomarkers such as IDH1, 1q-19p co-deletion, h3k27, TERT (Killela et al., 2014; Marton et al., 2019; Zhang Z. Y. et al., 2019), but the existed biomarkers still cannot fully predict the overall survival for all glioblastoma patients, such as IDH1 wild-type in WHO grade 2 gliomas or in recurrent gliomas; moreover, we know a little about of the MGMT demethylation status in glioma patients. Unlike many other types of malignant tumor, glioblastoma lacks of effective treatment measures and drug targets (Snape and Warr, 2015; Higashijima and Kanki, 2019; Ruta et al., 2019). Recent phase II/III clinical trials on glioblastoma were all failed, including immune checkpoint inhibitor PD-1 or PD-L1 (Berghoff and Preusser, 2016; Charoentong et al., 2017; Kurz et al., 2018) or anti-angiogenic drugs like bevacizumab (Kurz et al., 2018; Moriya et al., 2018). Life is composed of complicated regulator control system, the cancer happened normally involved in gene mutation, change of epigenetics and gain of fusion-gene (Liang et al., 2019). Thus, through integrating analysis of multi-omics data on glioblastoma is meaningful, which could systematically study the negative molecular event like genomic instability and somatic mutation (Song et al., 2019; Zhang Z. Y. et al., 2019). In this study, we performed integrated analysis via TCGA database of glioblastoma [(NIH), Genomic Data Commons database (GDC)^1^ ], aimed to complete a new molecular classification and provide some new treatment targets for GBM. As a result, we enrolled 117patients that all contained SNP, DNA copy, DNA methylation and mRNA expression profile data. After combined the multidimensional data with clinical information and cluster of clusters analysis steps, we divided theses GBM samples into two novel subtypes (HX-1 and HX-2), among the two subtypes, we identified 15 genes and 3 methylation variable position which are associated with overall survival, and the subtype HX-2 has an obvious higher mutation frequency than subtype HX-1, moreover, the NK cells activated rate in HX-2 is also higher than HX-1 group.

## Results

### Mutation Analysis Reveals

**FIGURE 1:**
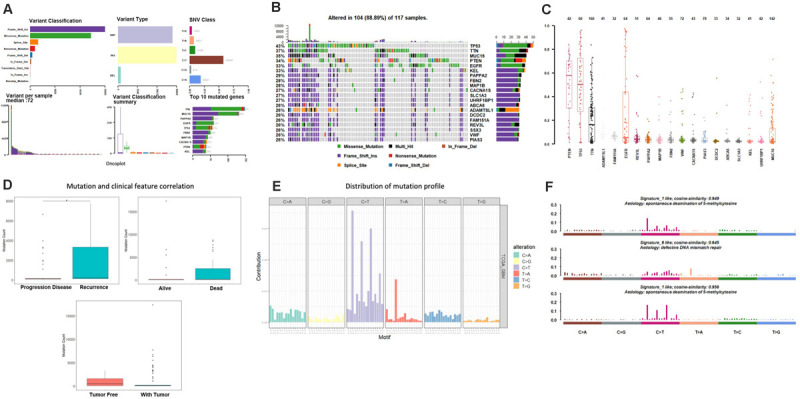
Mutation analysis for enrolled samples. **(A)** Tumor mutation profile of glioblastoma samples. **(B)** Oncoplot with the top 20 most mutated genes. **(C)** Frequency distribution of the top 20 gene mutations in glioblastoma. **(D)** Mutation and clinical feature correlation. **(E)** Distribution of mutation profile. **(F)** Mutation correlation of character and cosmic mutation signature.

As the first step, we performed statistical analysis for the enrolled 117GBM samples, annotated the mutation types, depicted proportion of different types of base changes and the top 10 mutation genes. Among these patients, the median age at initial diagnosis was 62(from 21 to 89), and 44 of them are female, more details of each patients could find in Supplementary Table 1. The overall description of the results is revealed in Figure 1A. In glioblastoma, the most common mutation type is C > T. Figures 1A,B have displayed the 20 most mutated genes and metadata information such as molecular subtypes information. Figure 1C disclosed the frequency distribution of the top20 gene mutations in GBM, the gene with the highest mutation rate is PTEN, 56% of samples had gene mutation on PTEN.

We separately counted the number of somatic mutations in each GBM sample and matched the clinical characteristics of these samples, the clinical features including survival status, tumor recurrence, etc. The analysis results indicated that the somatic mutations between tumor recurrence and progression of disease existed huge difference, and the recurrence samples has a higher number of mutations (Figure 1D).

Somatic mutations are widespread events in tumorigenesis, a few of gene mutations could directly cause tumor happening, and those genes are called driver genes (Higashijima and Kanki, 2019). We used MutSigCV to predict driver gene of the samples based on mutation data. When the significance threshold was *q* < 0.01, a total of 925 candidate genes were obtained.

Considering the mutation site of each sample and the bases at 1 bp position upstream and downstream of the mutation site, we divided the mutation into 96 types according to the upstream and downstream mutation site, calculated the frequency distribution of the 96 mutation types of the 117 sample (Figure 1E). Moreover, somatic mutations are present in all cells of the human body and occur throughout life. They are the consequence of multiple mutational processes, including the intrinsic slight infidelity of the DNA replication machinery, exogenous or endogenous mutagen exposures, enzymatic modification of DNA and defective DNA repair. Different mutational processes generate unique combinations of mutation types, termed “Mutational Signatures”^2^. In this study, to figure out the relationship between the mutation spectrum distribution of GBM samples and mutational signatures in *cosmic*, we subsequently conducted non-negative matrix factorization analysis based on 96 mutation types of the 117 sample, and extracted three somatic point mutations (Figure 1F). We found that the glioblastoma mutation spectrums are mainly related to signature_27 like, signature_1 like and signature_10 like.

### Copy Number Variation Analysis

**FIGURE 2:**
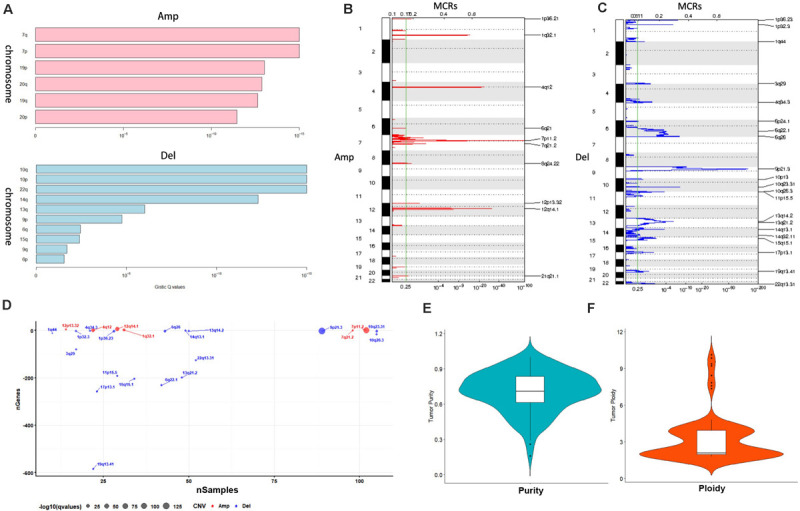
Copy number variation analysis. **(A)** GISTIC analysis for enrolled samples, upper panel: amplification of chromosome arm, lower panel: deletion of chromosome arm. **(B,C)** Distribution of minimal common regions (MCRs). **(D)** The number of genes in minimal common regions (the number of genes in the missing region is represented by a negative value). **(E,F)** Purity and ploidy analysis.

A total of 117 samples were conducted by GISTIC analysis. The results suggested that 7q,7p,19p amplification and 10q,10p, 22q deletion are most notable, and Figure 2A revealed the chromosome arms when GISTIC test significant (*Q*-value < 10^–5^). In all tumor samples, there were 10 amplifications and 21 copy number deletions in minimal common regions (MCRs), these MCRs are showed in Figures 2B,C, among them, the most significant amplification position are 7p11.2, 12q14.1, the most significant deletion position are 9p21.3,10q23.31. Figure 2D revealed the minimal common regions (MCRs) and the genes within the MRCs (the deletion genes in the region is represented by a negative value).

We then used ABSOLUTE software to evaluate the tumor purity and ploidy based on copy number variation (CNV), as showed in Figures 2E,F, the tumor purity ranged from 0.16–1, and the tumor cell genome ploidy was ranged from 1.82–10.13, which suggested that genomic disorder is a common event in tumorigenesis.

### Clustering by Integrated Platforms

**FIGURE 3:**
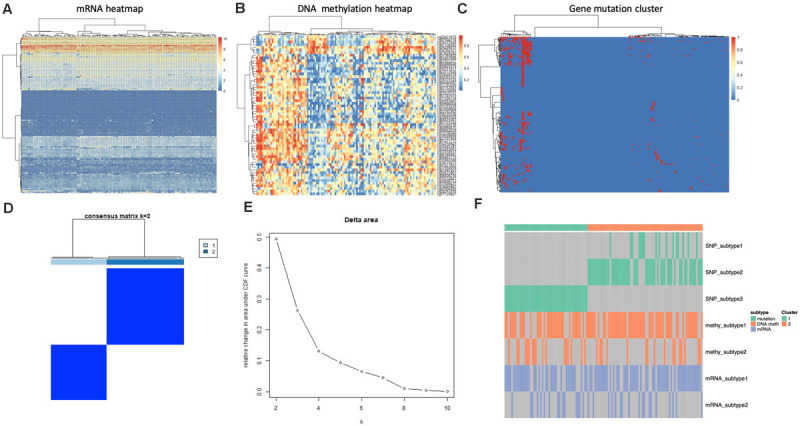
Identification of subtype with cluster of different platforms. **(A)** Clustering mRNA expression profile. **(B)** Clustering of DNA methylation. **(C)** Clustering of gene mutation. **(D,E)** CoCA cluster analysis, all samples can be divided into 2 subtypes, *K* = 2, HX-1(Cluster 1) and HX-2 (Cluster 2) subtype. **(F)** Subtype classification with single platform clustering results display.

We utilized four single platform data (SNP, DNA copy, DNA methylation,mRNA expression profile) to integrate with clinical information. When the significance threshold is set to 0.01(*q* < 0.01), 333 gene mutations, 60 DNA methylation sites, and 123 mRNAs are associated with prognosis of GBM patients; however, there were no significant CNV position with prognosis in our array. According to the expression of 123 mRNAs, the samples can be divided into 3 categories (Figure 3A). According to the information of 60 methylation sites, the samples can be divided into 2 subtypes (Figure 3B). According to the mutation information of 333 genes the samples can be divided into 2 subtypes (Figure 3C).

We next used CoCA cluster analysis method to conduct cluster analysis again, the data was derived from SNP, DNA methylation and mRNA platform, finally, we obtained two novel subtypes from all GBM samples, and we named these subtypes as HX-1 and HX-2 (Figure 3D). Figure 3E represents the delta area curve of consensus clustering, indicating the relative change in area under the cumulative distribution function (CDF) curve for each category number k compared with k-1. When the subtypes were classified into two groups (*K* = 2), the area under the cure is biggest. We also plot the information of the subtypes with each platform (Figure 3F). It suggests that HX1 and HX2 are more correlated to SNP1, SNP2, and SNP3, but not correlated to methylation subgroups or mRNA subgroups.

### Subtype Analysis

**FIGURE 4:**
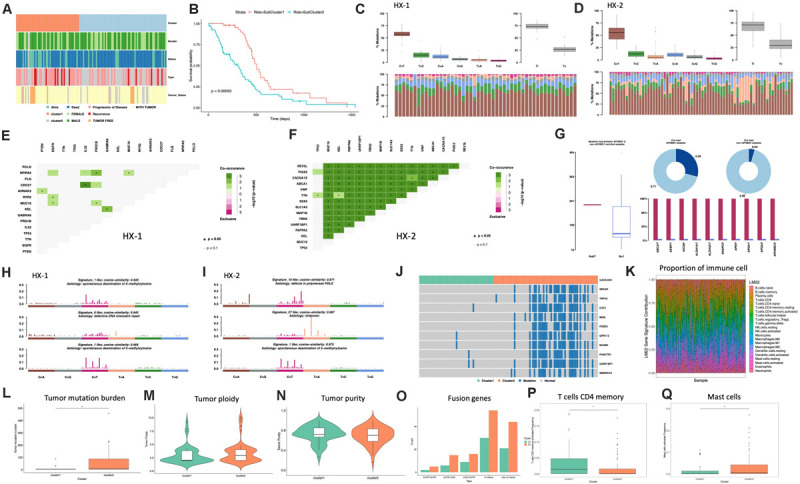
Analysis of subtypes. **(A)** Clinical characters of subtypes. **(B)** Survival curve between subtypes. **(C)** Distribution of mutation types of HX-1. **(D)** Distribution of mutation types of HX-2. **(E)** Mutually exclusive or co-occurring events in HX-1. **(F)** Mutually exclusive or co-occurring events in HX-2. **(G)** Cluster of APOBEC (“apolipoprotein B mRNA editing enzyme, catalytic polypeptide-like”) analysis of HX-2. **(H)** Mutation signatures of HX-1. **(I)** Mutation signatures of HX-2. **(J)** Distribution of significant difference genes between HX-1 and HX-2. **(K)** Distribution of immune cells. **(L)** Tumor mutation burden comparison. **(M,N)** Purity and ploidy analysis between HX-1 and HX-2. **(O)** Fusion genes events in HX-1 and HX-2. **(P)** Distribution of T cells CD4 memory activated between HX-1 and HX-2. **(Q)** Distribution of mast cells activated between HX-1 and HX-2.

Firstly, we analyzed the clinical features for each subtype, such as gender, tumor status, survival status and the median survival time etc. (Figure 4A and Table 1), the median survival time between each group (HX-1 and HX-2) has significant differences (*P* = 0.00053), the data indicated that the HX-2 had an obviously poor OS (Figure 4B), the median OS for HX-1 is 445 days, and the median OS for HX-2 is 231.5 days, *P*-value is 0.00053.

**TABLE 1:** Clinical features for each subtype.

	**HX-1**	**HX-2**	***P*-value**
Female	16	28	0.5072
Male	32	40	
Not available	1	0	
Alive	18	12	0.0285
Dead	30	56	
Not available	1	0	
Progression of disease	24	25	0.6217
Recurrence	8	5	
Not available	17	38	
Tumor free	6	4	0.4655
With tumor	39	53	
Not available	4	11	

We further want to identify whether each subtype differs in the type of mutation, as shown in Figures 4C,D, HX-1 and HX-2 were mainly happened as C > T mutation, and Ti (transition) frequency was higher than TV (transversion) frequency (Figures 4C,D).

Many genes that cause cancers often with mutually exclusive or co-occurring events, in order to determine which genes will happen with mutually exclusive or co-occurring events, we conduct Fisher’s exact test for any two gene mutations, and we found a plenty of gene co-occurring events in HX-2 subtype instead of HX-1 (Figures 4E,F).

APOBEC (“apolipoprotein B mRNA editing enzyme, catalytic polypeptide-like”) is a family of evolutionarily conserved cytidine deaminases. In humans, they help protect from viral infections. These enzymes, when misregulated, are a major source of mutation in numerous cancer types (Rebhandl et al., 2015). We used R package maftools to proceed APOBEC analysis. As shown in Figure 4G, only subtype HX-2 had APOBEC cluster samples, the genes with mutation rate which significantly high were revealed in Figure 4G, the box plot shows differences in mutation load between APOBEC-enriched and non-enriched samples, donut plots display the proportion of mutations in tCw context, bar plots show the top 10 differentially mutated genes between APOBEC-enriched and non-APOBEC- enriched samples.

We also compared the 96 signatures collected in cosmic with each subtype; as a result, the mutational signatures in each subtype were both associated with signature1 (Figure 4H), but the HX-1 had high similarity with signature6 subtype independently; the HX-2 subtype also had high similarity with signature10 and signature 27 (Figure 4I).

In order to identify the gene mutations for each subtype, we counted the total amount of mutations in each subgroup of each gene, and then conduct chi-square test. Finally, we identified 727 different mutations in reach subtypes, the subtype HX-2 had an obvious higher mutation rate than HX-1 (Figure 4J). We subsequent counted the tumor mutation burden (TBM) for each subtype, the result confirmed the TBM in HX-2 (TBM = 55.4) is significantly higher than HX-1 (TMB = 5.7, *P* = 3.881e-06, Figure 4L). There was no significant difference of each subtype on tumor ploidy (Figure 4M) and purity (Figure 4N).

We download fusion gene baseline from http://54.84.12.177/PanCanFusV2/database. In total, we identified 144 fusion genes in HX-1 cluster and 284 fusion gene in HX-2 (Figure 4O, Supplementary Table 2). We uploaded the expression data of 117 GBM samples to cibersort website, calculated the proportion of 22 immune cells in these samples (Figure 4K). Then, the distribution of the proportion of each immune cell between the two subgroups was calculated separately, we determined that proportion of T cells CD4 memory activated (Figure 4P) and mast cells activated (Figure 4Q) was significant different between HX-1 and HX-2.

### Prognostic Marker Identification and Validation

**FIGURE 5:**
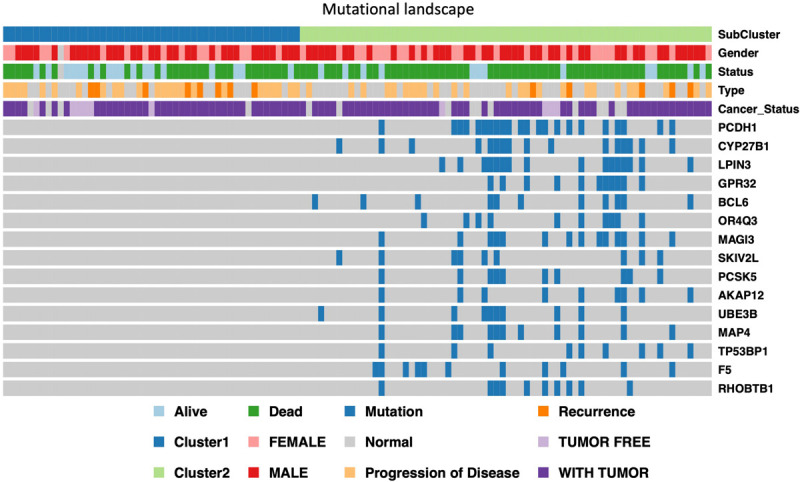
Display of landscape between HX-1 and HX-2, the clinical features and mutation status of 15 driver gene were integrated into subtypes, it can be clearly seen from the figure that the frequency of gene mutations in HX-2 subtype is much higher than that of HX-1.

In order to further identify of the prognostic markers for the subtypes, we conjointly analyzed the 19 DE genes, 27 DE methylation position and 727 DE genes between HX-1 and HX-2. The analysis results show that when the significance threshold is set to 0.05, there had three methylation positions [cg16957313(DUSP1), cg17783509(PHOX2B),cg23432345(HOXA7)] and 21 genes were associated with prognosis, in which 15 genes were same as in Mut2SigCV analysis. In addition, the distribution of all GBM cases based on TCGA is displayed in Supplementary Figure 1 according to the mutation signature of these 15 genes. The survival curve of these 21 associated prognosis factors were showed in Supplementary Figure 2 We also described the landscape of the 15 genes between the two subtypes (Figure 5).

Moreover, to validate the outcome of our analysis, the 15 mutant genes mutation signature genes used to develop a cancer-related risk signature. Samples from the Chinese Glioma Genome Atlas (CGGA) dataset were divided into high risk group and low risk group. These samples carrying mutations within 15 genes were defined as high-risk group (*n* = 11) in CGGA primary GBM cohort; while the others were defined as low-risk group (*n* = 42). According to the Kaplan-Meier survival analysis, the prognosis of high-risk group was strikingly worse than that of low-risk group (Supplementary Figure 3A, *P* = 0.032). Moreover, the 15 gene signature in the CGGA primary GBM cohort showed a high area under the receiver operating characteristic curve (AUC = 0.632) (Supplementary Figure 3B), close to that in the TCGA GBM cohort (AUC = 0.756) (Supplementary Figure3C).

## Discussion

Glioblastomas (GBM) is the most invasive and prevalent types of glioma with extremely poor prognosis and limited treatment options (Rebhandl et al., 2015). In recent years, tremendous articles reported the molecular characterization of GBM, make us better understanding of how to use the key molecules to predict the OS for glioma patients (Colaprico et al., 2016; Holdhoff, 2018; Higashijima and Kanki, 2019; Marton et al., 2019). However, most of the published articles were based on single platform analysis, which is hard to explain why the similar molecular pattern may induce diverse prognosis in GBM patients sometimes. In order to make a comprehensive understanding on molecular characteristic of GBM, we used the unsupervised clustering method to cluster the data from four different platforms (SNP, DNA copy, DNA methylation,mRNA expression) and subsequently used the cluster of clusters analysis (CoCA) method to further identify the subtypes of GBM. Therefore, through systematic studying of the integrated multi-omics analysis, genomic instability, somatic mutation and the molecular characteristics of each GBM subgroup, we hope we can provide new ideas and novel theoretical basis for early diagnosis and individualized treatment for GBM patients.

We conducted the SNP associated analysis in the first step, our result showed the glioblastoma was characterized by prominence of C > T. The signatures of mutational processes in human cancer was firstly reported by Michael R. Stratton and his colleagues, they concluded more than 20 distinct mutational signatures from 4,938,362 mutations from 7,042 cancers (Wu et al., 2019). We extracted the mutation characteristics of somatic point mutations, the result showed that the mutation spectrum of glioblastoma is similar to signature27,signature1 and signature10 which collected in cosmic. As reported, the Signature 1A/B is probably related to the relatively elevated rate of spontaneous deamination of 5-methyl-cytosine which results in C > T transitions and which predominantly occurs at NpCpG trinucleotides, and signature10 was the associated with altered activity of the error-prone polymerase Pol ε consequent on mutations in the gene. However, the reason for signature 27 is still unknown.

We next use clusters analysis (CoCA) method to classified the subtype of enrolled glioblastoma samples as HX-1 and HX-2, the main mutation signature of the two subtypes are the same as C > T, however, there were a plenty of gene co-mutation events in HX-2 but not shown in HX-1, the Tumor mutation burden in HX-2 was significant higher that HX-1, and the median survival forHX-2 is 231.5 days, much shorter than HX-1 445 days, suggested that the HX-2 subtype is more aggressive than HX-1 subtype, and HX-2 occurred from high frequency of gene mutation.

The proportion of *T cells CD4 memory activated* and *mast cells activated* were determined significant difference between HX-1 and HX-2 in our result. Dongrui Wang et al. found that maintenance of the CD4 + subset was positively correlated with the recursive killing ability of CAR T cell products derived from GBM patients (Alexandrov et al., 2013). His finding identified CD4 + CAR T cells as a highly potent and clinically important T cell subset for effective CAR therapy. This may probably explain why the HX-1 had the better prognosis. Moreover, recent research indicated that mast cells (MCs) upon activation by glioma cells produce soluble factors including IL-6, which are documented to be involved in cancer-related activities and promoted glioma cell differentiation and growth (Wang et al., 2018). It was also figured out that MCs exert their effect via inactivation of STAT3 through GSK3β downregulation. This could probably explain why the HX-2 cluster had the shorter OS.

We further analyzed the negatively regulative biomarkers which may distinguish the OS of HX-2 from HX-1, and we identified 3 methylation variable positions [cg16957313(DUSP1), cg17783509(PHOX2B), cg23432345(HOXA7)] and 15 genes (PCDH1, CYP27B1, LPIN3, GPR32, BCL6, OR4Q3, MAGI3, SKIV2L, PCSK5, AKAP12, UBE3B, MAP4, TP53BP1, F5, RHOBTB1) that may induce poor overall survival for HX-2. Some of these genes have been reported to be associated with the malignant behavior of glioblastoma. For example, studies have shown that BCL6 is essential for the survival of GBM cells (Attarha et al., 2017), the overexpression of BCL6 is associated with poor prognosis for glioma patients, BCL6 gene could inhibits the expression of wild-type p53 and its target genes in GBM cells. In gliomas, the expression levels of MAGI3 and PTEN were reported significantly down-regulated, and for glioma C6 cell line, overexpressed MAGI3 will inhibits Akt phosphorylation, and inhibits cell proliferation (Xu et al., 2017). We also identified some novel genes which are still not been reported, such as PCDH1, LPIN3, GPR32, SKIV2L, PCSK5.

In this study, we used a comprehensive bioinformatics method to integrate 4 platform data of glioblastoma, and further identified two novel subtypes of glioblastoma which could be characterized by the cluster of 3 methylation variable position and 15 gene mutation, the multi-omic signatures for the prognosis of glioblastoma developed by us were also be validate in CGGA independent dataset. We hope that our research could provide potential stratification marker for clinical outcome and new theoretical basis for glioblastoma.

## Materials and Methods

### TCGA Data Acquisition

The TCGAbiolinks R package was used to help us obtain patients data from the National Institutes of Health (NIH), Genomic Data Commons database (GDC)^3^ (Holdhoff, 2018). Briefly, we get 577 SNP6 Copy Number segment GBM samples data and 411 samples methylation microarrays data from the website http://firebrowse.org/, and we also downloaded 154 GBM samples mRNA expression data from https://portal.gdc.cancer.gov/. After filtrate these data and link sample information, there are 117 sample contained multi-omics data, which means all the filtered samples contained gene mutation data, CNV data, methylation data and mRNA expression data. Our subsequent analysis was based on these data. The fusion gene result subsequently used was acquired from TUMOR FUSION GENE DATA PORTAL database^4^.

### Single Nucleotide Polymorphism (SNP) Analysis

MutSigCV module in GenePattern was used to analysis the driver gene in GBM^5^ (Ma et al., 2015). There are strong correlations between somatic mutation frequencies in cancers and both gene expression level and replication time of a DNA region during the cell cycle, MutsigCV analysis could substantially reduce the number of false positives, especially when applied to tumor samples that have high mutation rates.

We use the maftools R package^6^ (Lawrence et al., 2013) and SomaticSignatures^7^ (Mayakonda et al., 2018) to conduct mutation analysis and plot the mutation spectrum and characteristics.

### Copy Number Variation Analysis

GISTIC module in GenePattern was also used to extract the landmark CNV events in GBM, the parameters in GISTIC algorithm were set as follows: *Q*-value < 0.05 as statistics significance, confidence levels were set as 95% to confirm peak region. Chromosome arm length was set as 0.98 when analyzed the chromosome arm mutation. Tumor purity and ploidy character were analyzed by the R package R ABSOLUTE^8^.

### Subtype Identification of Glioblastoma

Unsupervised clustering was proceeded based on the three different platforms (SNP, DNA methylation, and mRNA expression profile) and molecules associated of overall survival, the we conduct the clustering again based on a method called cluster of clusters analysis (CoCA) (Hoadley et al., 2014; Gehring et al., 2015). Briefly, Subtype calls from each of the 4 platforms analyzed for subtypes within each data type were used to identify relationships between the different classifications. Subtypes defined from each platform were coded into a series of indicator variables for each subtype. The matrix of 1 and 0s was used in ConsensusClusterPlus R package (Gehring et al., 2015) to identify structure and relationship of the samples. Parameters for Consensus cluster were 80% sample resampling with 1000 iterations of hierarchical clustering based on a Pearson correlation distance metric. and ultimately, we acquired the two subtypes result from glioblastoma that integrated the data of different platforms. We named these two subtypes as subtype HX-1 and subtype HX-2.

### Characteristic Analysis of Subtypes

Chi-square test was used to the characteristic analysis of GBM subtypes, including survival state and progression of disease.

R package limma^9^ (Smaglo et al., 2015) was conduct to screen the valuable biomarkers within the subgroups, we tried to filter the difference expressed (DE) mRNA and methylation variable positions (MVPs), and finally proceed KEEG and GO analysis for those DE mRNA and MVPs.

We also utilized the maftools to map the gene mutation characteristic in GBM subtypes, including C>T, T>C, C>A, T>G, C>G, T>A, Ti(transition) and TV (transversion). Moreover, mutation signature analysis and APOBEC enrichment analysis (apolipoprotein B mRNA editing enzyme, catalytic polypeptide-like) were also conduct between the subtypes.

### Infiltrated Immune Cells Estimate

Tumor immune cell infiltration refers to the migration of immune cells from the blood to the tumor tissue and begins to exert its effects. The infiltration of immune cells in tumor directly affects the overall survival in GBM patients. Thus, to quantify the proportion of immune cells in the enrolled samples, we used CIBERSORT algorithm (Ritchie et al., 2015; Chen et al., 2018; Zhang L. et al., 2019) and LM22 algorithm (Charoentong et al., 2017), and calculated the percentage of 22 types of human immune cells in GBM, concluding the B cells, T cells, natural killer cells, macrophages and dendritic cells.

## Data Availability Statement

The datasets presented in this study can be found in online repositories. The names of the repository/repositories and accession number(s) can be found in the article Supplementary Material.

## Author Contributions

All authors listed have made a substantial, direct and intellectual contribution to the work, and approved it for publication.

## Conflict of Interest

The authors declare that the research was conducted in the absence of any commercial or financial relationships that could be construed as a potential conflict of interest.

## Footnotes

1 https://gdc.cancer.gov/

2 https://cancer.sanger.ac.uk/cosmic/signatures

3 https://gdc.cancer.gov/

4 https://tumorfusions.org/PanCanFusV2/

5 https://cloud.genepattern.org/gp/pages/index.jsf

6 https://bioconductor.org/packages/release/bioc/html/maftools.html

7 https://bioconductor.org/packages/release/bioc/html/SomaticSignatures.html

8 https://software.broadinstitute.org/cancer/cga/absolute_download

9 https://bioconductor.org/packages/release/bioc/html/limma.html

## Supplementary Material

Supplementary Figure 1 Mutation signature of these 15 DE genes.

Supplementary Figure 2 Survival curves of the three methylation variable position [cg16957313(DUSP1), cg17783509(PHOX2B), cg23432345(HOXA7)] and 15 genes (PCDH1, CYP27B1, LPIN3, GPR32, BCL6, OR4Q3, MAGI3, SKIV2L, PCSK5, AKAP12, UBE3B, MAP4, TP53BP1, F5, RHOBTB1).

Supplementary Figure 3 Prognostic marker validation. **(A)** Kaplan–Meier survival analysis of Chinese Glioma Genome Atlas (CGGA) dataset. **(B)** Operating characteristic curve (AUC = 0.632) of CGGA primary GBM cohort. **(C)** Operating characteristic curve (AUC = 0.756) of TCGA GBM cohort.
