# Supplementary figures and images for: Multi-Omics Analysis Reveals Novel Subtypes and Driver Genes in Glioblastoma
Source: Front Genet. 2020 Nov 26;11:565341. doi: 10.3389/fgene.2020.565341 (PMC7726196; doi:10.3389/fgene.2020.565341)

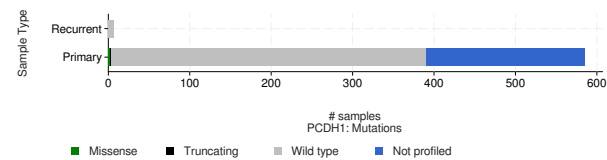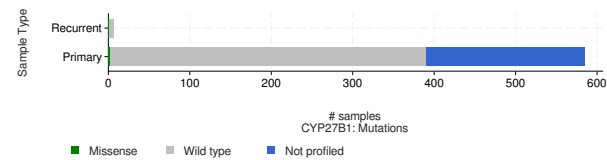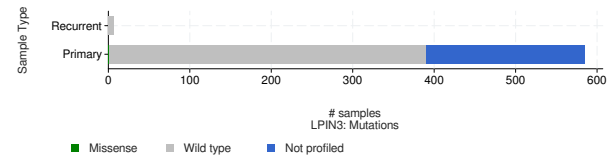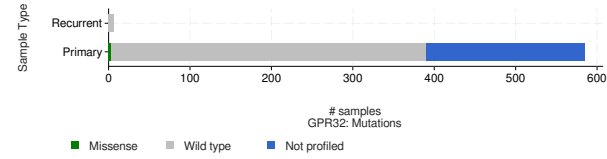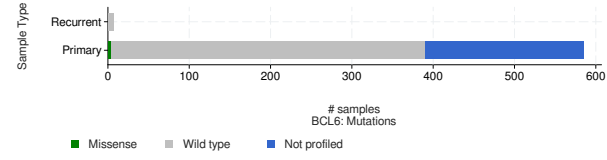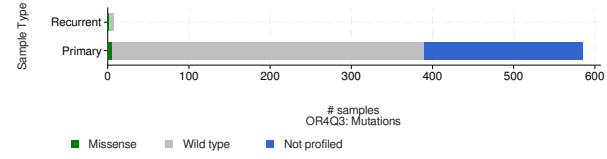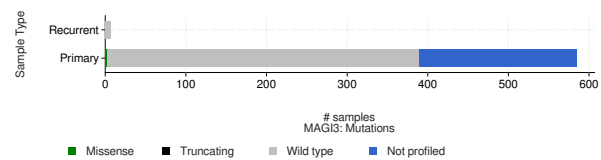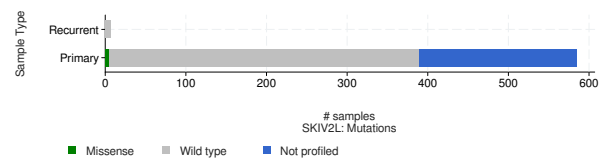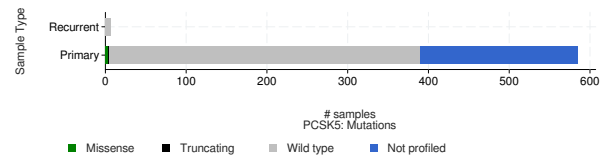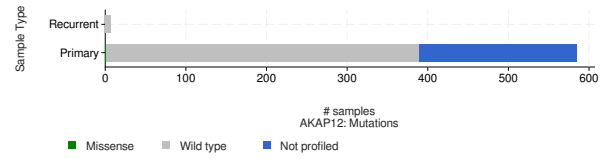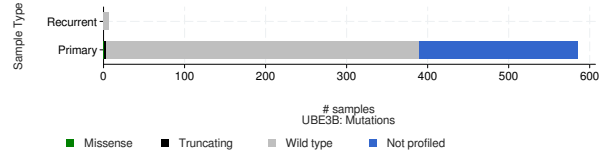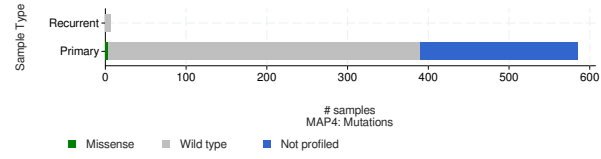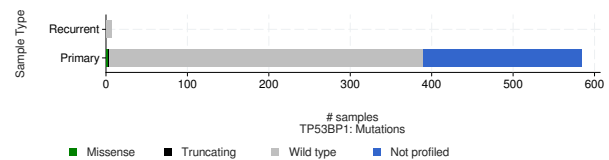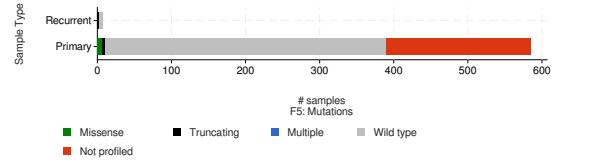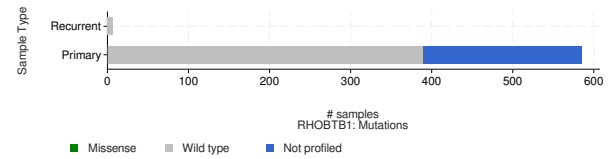

Supplement: Supplementary Figure 1 — Mutation signature of these 15 DE genes. [file Presentation_1.pdf]

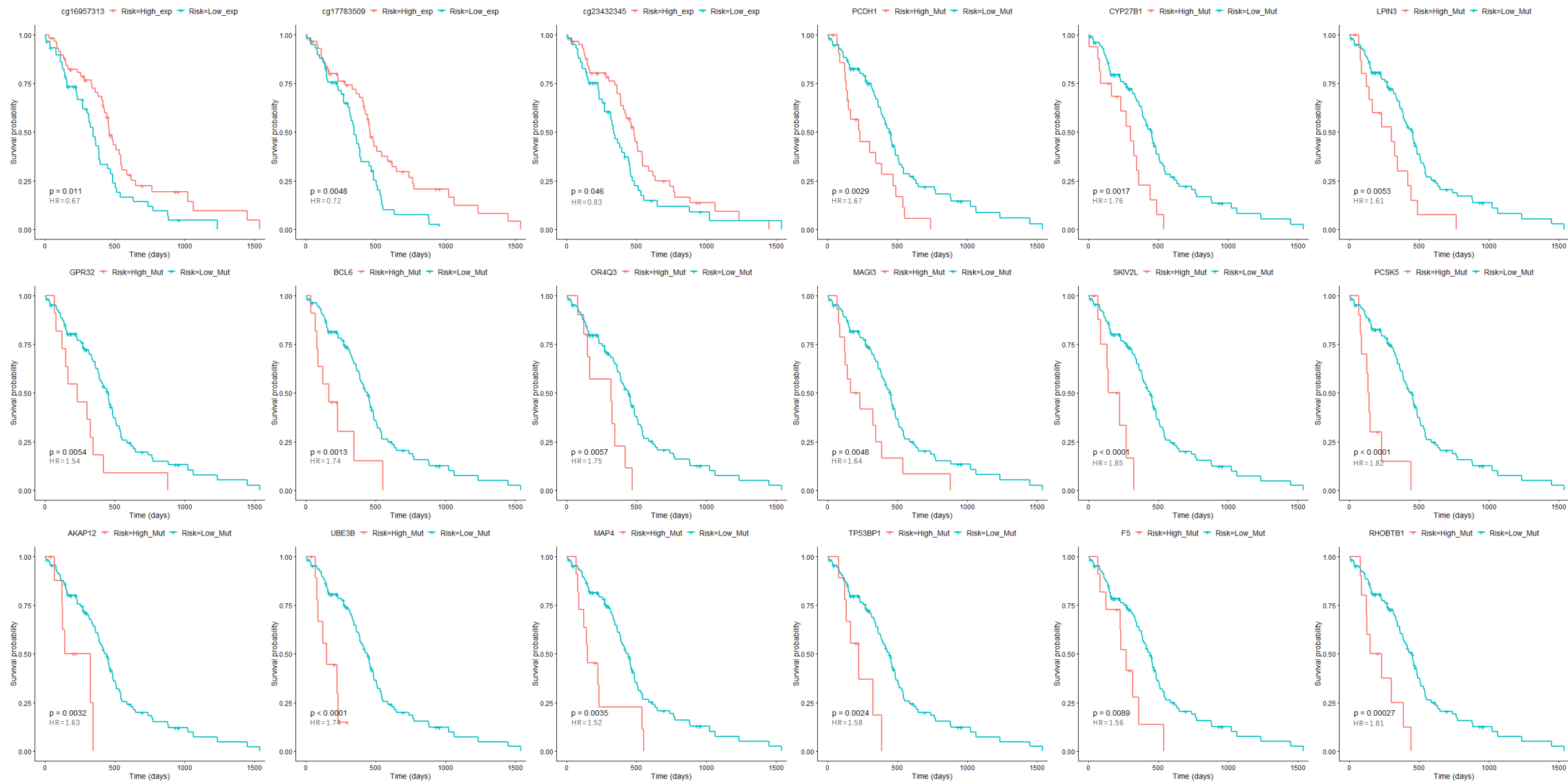

Supplement: Supplementary Figure 2 — Survival curves of the three methylation variable position [cg16957313(DUSP1), cg17783509(PHOX2B), cg23432345(HOXA7)] and 15 genes (PCDH1, CYP27B1, LPIN3, GPR32, BCL6, OR4Q3, MAGI3, SKIV2L, PCSK5, AKAP12, UBE3B, MAP4, TP53BP1, F5, RHOBTB1). [file Presentation_2.pdf]

**A****CGGA set (n=53)**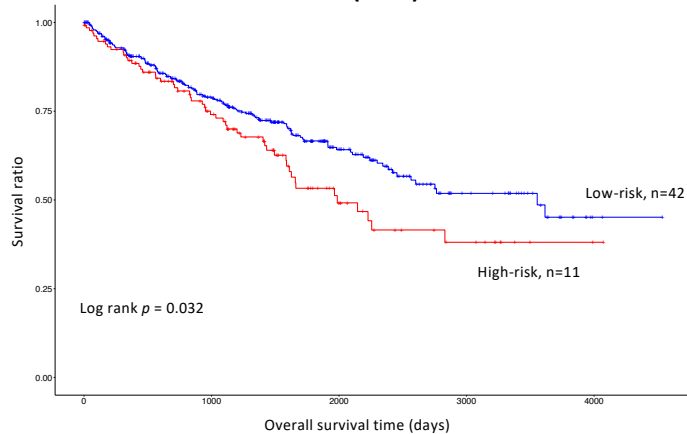**B****CGGA AUC: 0.632**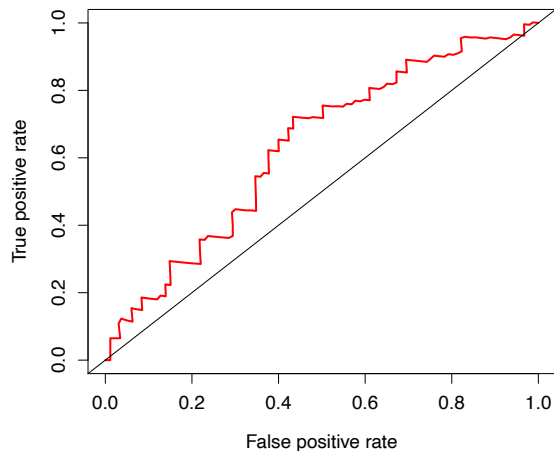**C****TCGA AUC: 0.756**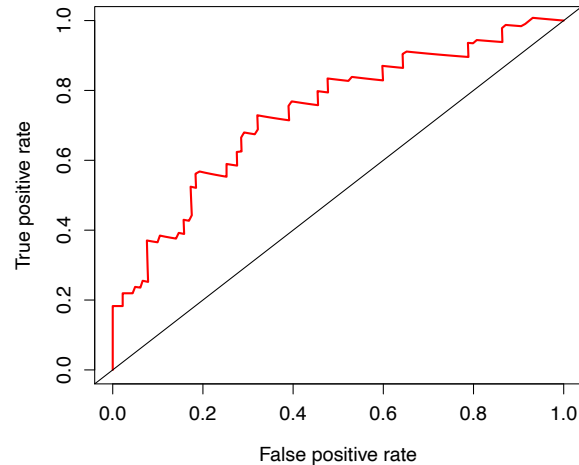

Supplement: Supplementary Figure 3 — Prognostic marker validation. (A) Kaplan–Meier survival analysis of Chinese Glioma Genome Atlas (CGGA) dataset. (B) Operating characteristic curve (AUC = 0.632) of CGGA primary GBM cohort. (C) Operating characteristic curve (AUC = 0.756) of TCGA GBM cohort. [file Presentation_3.pdf]
